# Supplementary material for: Modeling glioblastoma heterogeneity as a dynamic network of cell states
Source: Mol Syst Biol. 2021 Sep 16;17(9):e10105. doi: 10.15252/msb.202010105 (PMC8444284; doi:10.15252/msb.202010105)
Supplement: Supplementary file 6 — Source Data for Figure 5 [file MSB-17-e10105-s004.zip › Figure5A_sourcedata/GSEA_3071/nosigdb_stateII.GseaPreranked.1622795151171/RICHARDS_NEFTEL_CELL_2019_G2_M.html]

Details for gene set RICHARDS\_NEFTEL\_CELL\_2019\_G2\_M[GSEA]

|  || Dataset | state23071 |
| Phenotype | NoPhenotypeAvailable |
| Upregulated in class | na\_pos |
| GeneSet | RICHARDS\_NEFTEL\_CELL\_2019\_G2\_M |
| Enrichment Score (ES) | 0.6812994 |
| Normalized Enrichment Score (NES) | 3.2800295 |
| Nominal p-value | 0.0 |
| FDR q-value | 0.0 |
| FWER p-Value | 0.0 |
Table: GSEA Results Summary

  

Fig 1: Enrichment plot: RICHARDS\_NEFTEL\_CELL\_2019\_G2\_M      
 Profile of the Running ES Score & Positions of GeneSet Members on the Rank Ordered List

  

| PROBE | GENE SYMBOL | GENE\_TITLE | RANK IN GENE LIST | RANK METRIC SCORE | RUNNING ES | CORE ENRICHMENT || 1 | CCNA2 |  |  | 53 | 1.230 | 0.0326 | Yes |
| 2 | KIF2C |  |  | 73 | 1.132 | 0.0699 | Yes |
| 3 | KIFC1 |  |  | 88 | 1.094 | 0.1071 | Yes |
| 4 | TMPO |  |  | 99 | 1.056 | 0.1438 | Yes |
| 5 | CDCA8 |  |  | 113 | 1.018 | 0.1784 | Yes |
| 6 | KIF11 |  |  | 115 | 1.014 | 0.2158 | Yes |
| 7 | TACC3 |  |  | 130 | 0.992 | 0.2491 | Yes |
| 8 | CKAP2L |  |  | 148 | 0.959 | 0.2805 | Yes |
| 9 | H2AFZ |  |  | 213 | 0.864 | 0.2968 | Yes |
| 10 | CDCA3 |  |  | 219 | 0.858 | 0.3274 | Yes |
| 11 | BUB1B |  |  | 223 | 0.851 | 0.3583 | Yes |
| 12 | RACGAP1 |  |  | 241 | 0.831 | 0.3849 | Yes |
| 13 | TUBB4B |  |  | 271 | 0.784 | 0.4069 | Yes |
| 14 | TUBA1C |  |  | 277 | 0.779 | 0.4346 | Yes |
| 15 | SMC4 |  |  | 279 | 0.777 | 0.4632 | Yes |
| 16 | KIF4A |  |  | 309 | 0.746 | 0.4837 | Yes |
| 17 | ARHGAP11A |  |  | 346 | 0.712 | 0.5013 | Yes |
| 18 | AURKA |  |  | 360 | 0.705 | 0.5242 | Yes |
| 19 | TTK |  |  | 365 | 0.701 | 0.5492 | Yes |
| 20 | KPNA2 |  |  | 420 | 0.669 | 0.5608 | Yes |
| 21 | FOXM1 |  |  | 423 | 0.667 | 0.5850 | Yes |
| 22 | NCAPD2 |  |  | 457 | 0.647 | 0.6009 | Yes |
| 23 | ECT2 |  |  | 510 | 0.626 | 0.6113 | Yes |
| 24 | CENPF |  |  | 575 | 0.588 | 0.6174 | Yes |
| 25 | CCNB1 |  |  | 579 | 0.587 | 0.6385 | Yes |
| 26 | CKAP2 |  |  | 619 | 0.569 | 0.6500 | Yes |
| 27 | SPAG5 |  |  | 642 | 0.555 | 0.6651 | Yes |
| 28 | GPSM2 |  |  | 719 | 0.526 | 0.6660 | Yes |
| 29 | RANGAP1 |  |  | 736 | 0.520 | 0.6813 | Yes |
| 30 | CDC20 |  |  | 844 | 0.486 | 0.6730 | No |
| 31 | PLK1 |  |  | 1009 | 0.442 | 0.6490 | No |
| 32 | BUB1 |  |  | 1145 | 0.408 | 0.6309 | No |
| 33 | MZT1 |  |  | 1219 | 0.393 | 0.6275 | No |
| 34 | ANP32E |  |  | 1334 | 0.371 | 0.6132 | No |
| 35 | KNSTRN |  |  | 1458 | 0.346 | 0.5958 | No |
| 36 | REEP4 |  |  | 1786 | 0.300 | 0.5263 | No |
| 37 | OIP5 |  |  | 1789 | 0.299 | 0.5370 | No |
| 38 | MXD3 |  |  | 2156 | 0.256 | 0.4563 | No |
| 39 | ARL6IP1 |  |  | 3574 | -0.586 | 0.1291 | No |
Table: GSEA details [plain text format]

  

Fig 2: RICHARDS\_NEFTEL\_CELL\_2019\_G2\_M: Random ES distribution      
 Gene set null distribution of ES for **RICHARDS\_NEFTEL\_CELL\_2019\_G2\_M**
